# Association between different levels of dysglycemia and metabolic syndrome in pregnancy

**DOI:** 10.1186/1758-5996-1-3

**Published:** 2009-08-26

**Authors:** Carlos A Negrato, Lois Jovanovic, Alex Rafacho, Marcos A Tambascia, Bruno Geloneze, Adriano Dias, Marilza VC Rudge

**Affiliations:** 1 School of Medicine of Botucatu, São Paulo State University - UNESP, Botucatu, São Paulo, Brazil; 2 Sansum Diabetes Research Institute, Santa Barbara, California, USA; 3 School of Sciences, São Paulo State University - UNESP, Bauru, São Paulo, Brazil; 4 School of Medical Sciences, State University of Campinas - UNICAMP, Campinas, São Paulo, Brazil

## Abstract

**Background:**

In this study, we sought to evaluate the prevalence of metabolic syndrome (MS) in a cohort of pregnant women with a wide range of glucose tolerance, prepregnancy risk factors for MS during pregnancy, and the effects of MS in the outcomes in the mother and in the newborn.

**Methods:**

One hundred and thirty six women with positive screening for gestational diabetes mellitus (GDM) were classified by two diagnostic methods: glycemic profile and 100 g OGTT as normoglycemic, mild gestational hyperglycemic, GDM, and overt GDM. Markers of MS were measured between 24-28^th ^during the screening.

**Results:**

The prevalence of MS was: 0%; 20.0%; 23.5% and 36.4% in normoglycemic, mild hyperglycemic, GDM, and overt GDM groups, respectively. Previous history of GDM with or without insulin use, BMI ≥ 25, hypertension, family history of diabetes in first degree relatives, non-Caucasian ethnicity, history of prematurity and polihydramnios were statistically significant prepregnancy predictors for MS in the index pregnancy, that by its turn increased the adverse outcomes in the mother and in the newborn.

**Conclusion:**

The prevalence of MS increases with the worsening of glucose tolerance; impaired glycemic profile identifies pregnancies with important metabolic abnormalities even in the presence of a normal OGTT, in patients that are not classified as having GDM.

## Background

Short and long-term important consequences for the fetus, the newborn and the mother can occur when gestational diabetes mellitus (GDM) is present [1,2]. Pregnant women that show mild hyperglycemia, but do not meet the criteria for GDM diagnosis can still present glucose-mediated macrosomia, the same perinatal mortality rate and adverse pregnancy and perinatal outcomes as those with GDM [3-11].

Typically, a condition of insulin resistance develops in the second and third trimester of pregnancy. The impairment of insulin sensitivity makes pregnancy a diabetogenic condition. Nonetheless, only 3 to 5% of women develop GDM [12,13]. As it happens in type 2 diabetes mellitus (DM2) and in metabolic syndrome (MS), when dysglycemia is present, GDM is associated to both insulin resistance and impaired insulin secretion [14-16]. DM2, MS and GDM also share the same risk factors and have a corresponding prevalence within a given population and the same genetic susceptibility [17]. Many of the known metabolic components of the MS are predictive of GDM, which could be considered as one phase of the MS. MS is referred to the association of hyperinsulinemia, insulin resistance, visceral obesity, dyslipidemia, hypertension, and DM2 or impaired glucose tolerance [18]. These features increase the risk of atherosclerosis and coronary heart disease [19,20]. It is not known whether mild degrees of hyperglycemia are also associated to the components of MS and adverse pregnancy outcomes.

Thus, the aims of this study were to evaluate the prevalence of MS through the presence of its physical and clinical components in a cohort of pregnant women with a wide range of glucose tolerance and to analyze prepregnancy risk factors for the development of MS during pregnancy and its adverse impacts on pregnancy outcomes.

### Research design, patients and methods

This study was approved by the Institutional Review Board of the School of Medicine of Botucatu - São Paulo State University - UNESP, Brazil; where it was conducted from April 2004 through November 2005. One hundred and thirty six women with singleton pregnancies were assigned to participate if they presented a fasting glycemia level ≥ 90 mg/dl and/or risk factors for developing GDM. Between the 24^th ^through 28^th ^weeks of gestation, a 100 g OGTT and a glycemic profile were performed. The cutoff values for the OGTT were those proposed by Carpenter & Coustan (fasting ≥ 95 mg/dl; 1 hour ≥ 180 mg/dl; 2 hours ≥ 155 mg/dl; 3 hours ≥ 140 mg/dl) [21] and for the glycemic profile those proposed by Gillmer et al. (fasting ≥ 90 mg/dl and 1 hour post prandial ≥ 130 mg/dl) [22]. The glycemic profile was performed a week after the OGTT. Patients were taught on how to measure their glycemic levels using a glucometer in the fasting state at 8:00 o'clock AM, then post-prandial at 10:00 o'clock AM, midday, 2, 4 and 6 o'clock PM. If the results were borderline (10 mg/dl higher or lower than the cutoff values) they were repeated. After these procedures they were classified in four groups:

IA Group - Normal OGTT and glycemic profile (normoglycemic or control group)

IB Group - Normal OGTT and abnormal glycemia profile (mild hyperglycemic group)

IIA Group - Abnormal OGTT and normal glycemic profile (gestational diabetes group)

IIB Group - Abnormal OGTT and glycemic profile (overt gestational diabetes group)

Maternal characteristics such as age, parity, ethnicity, educational level, family income (Brazilian minimum wage), weight and length at birth, weight and prepregnancy body mass index (BMI), family's history of diabetes, hypertension, obesity, and dyslipidemia were recorded. At screening, weight, height, legs length, blood pressure, waist circumference, and hip circumference were measured. Obesity was defined as a prepregnancy body mass index (BMI) ≥ 30 kg/m^2^. Hypertension was considered when a systolic blood pressure >140 mmHg or a diastolic blood pressure > 90 mmHg, on at least two occasions at least six hours apart, was detected [23].

Blood samples were collected at the time the OGTT was performed to determine fasting levels of glucose, HbA_1c_, insulin, total, HDL-cholesterol, LDL- cholesterol, VLDL-cholesterol, and triglycerides. A 75 g OGTT was performed six weeks after delivery in those patients that presented glucose intolerance during pregnancy to check if they had returned or not to a normal glucose tolerant state.

All glucose determinations were conducted using glucose oxidase method (Glucose-analyzer II Beckman, Fullerton, CA, USA). Home blood glucose monitoring was performed with an Accu-chek Advantage II Glucometer (Roche Diagnostics GmbH, Mannheim, Germany). Total, LDL-cholesterol, HDL-cholesterol, VLDL-cholesterol, and triglycerides were measured by enzymatic colorimetric assay (Vitros 250, Ortho-Clinical Diagnostics, Rochester, New York, USA). HbA_1c _was determined by HPLC (high performance liquid column) method (Dia-Stat analyzer, Bio-Rad Laboratories, Hercules, CA, USA), and insulin using a specific radioimmunoassay kit (Linco Research, St. Charles, MO) with an intra and an interassay variation of 2.2 to 4.4% and 2.9 to 6.0%, respectively. Laboratory quality standards were routinely evaluated.

Newborn data collected included birth weight, length, ponderal index, gender, gestational age at delivery, Apgar scores, perinatal morbidity, and congenital malformations. Births were defined as preterm if gestational age was < 37 weeks. Ponderal index was calculated obtaining the ratio between 100 times the weight (in grams) and the cube of the length (in cm). The relation of newborns weight to gestational age was done according to Lubchenco's classification [24].

As there is no definition for MS in pregnancy, we adopted the following: any one of the two primary criteria [impaired glycemic profile and/or impaired OGTT, plus at least two of the following secondary criteria: hypertension (systolic blood pressure > 140 mmHg or a diastolic blood pressure > 90 mmHg on at least two occasions at least six hours apart); dyslipidemia (plasma triglycerides ≥ 2 SD above the mean of the control group and/or low HDL-Cholesterol < 39 mg/dl) and obesity (BMI ≥ 30 Kg/m^2 ^and/or waist ≥ 2 SD above the mean of pregnant women belonging to the control group)] [25-28].

The homeostasis model assessment (HOMA) was calculated to determine the degree of β-cell function (%) and insulin sensitivity (or resistance) (%S), using a computer program (HOMA2 model) that analyzes quantitatively these data [29].

### Statistical analysis

Data were presented as mean ± standard deviation (SD) or percentages. Analysis of variance (ANOVA) with Tukey post-test was performed to compare means for continuous variables with normal distribution. Kruskal-Wallis with Dunn post-test was performed when the supposition of homocedasticity and normality of distribution were not attempted. Fisher's exact test or Chi-square test plus Odds ratio (with 95% CI) was performed for proportions comparisons. The significance level adopted for all tests was < 0.05.

## Results

One hundred and thirty six patients were enrolled in the study. All of them completed the protocol. Data were available from 46 control group patients (IA group), 40 mild hyperglycemic (IB group), 17 GDM (IIA group) and 33 overt GDM (IIB group).

Women with overt diabetes were older (*p *< 0.002), had lower education levels (*p *< 0.001), lower family income (*p *< 0.001), were about 5 cm shorter (*p *< 0.001), and had approximately 3 cm shorter legs (*p *= 0.014) than those from the normoglycemic group. At birth, the overt GDM women were shorter (*p *= 0.05) and weighted less (*p *= 0.042) than control group. Prepregnancy, they had higher BMI (*p *= 0.001), larger waist (*p *< 0.001), higher systolic (*p *= 0.003) and diastolic blood pressure (*p *= 0.003). At screening, they had higher BMI (*p *< 0.001), larger waist (*p *< 0.001), larger waist-to-hip ratio (*p *= 0.002), higher systolic (*p *= 0.002) and diastolic (*p *= 0.02) blood pressure, higher fasting glucose (*p *< 0.001), HbA_1c _(*p *< 0.001), triglycerides (*p *= 0.016), VLDL (*p *= 0.037), and lower HDL (*p *= 0.014) cholesterol. Women with mild hyperglycemia (IB group) also had lower education level (*p *< 0.001) and shorter legs (*p *= 0.014) than those from the control group (Tables 1 and 2).

**Table 1:** Sociodemographic and clinical characteristics of the cohort according to glucose tolerance status (IA = normal glucose tolerance; IB = Impaired glycemic profile; IIA = Impaired 100 g OGTT; IIB = Impaired 100 g OGTT and glycemic profile).

***Variables***	***Groups***				***p-value***
	***IA***	***IB***	***IIA***	***IIB***	
Number	46	40	17	33	
Age (years)	28.7 ± 5.2	28.8 ± 6.4	31.4 ± 5.0	33.1 ± 5.6	0.002^(1)^
White (%)	89.1	77.5	82.4	60.6	0.025^(2)^
Education level (school years)	12.0 ± 3.6	10.7 ± 4.7	9.5 ± 4.6	7.1 ± 5.4	<0.001^(3)^
Family income	9.7 ± 5.4	7.7 ± 5.8	8.3 ± 5.4	3.5 ± 3.6	<0.001^(4)^
Diabetes in first degree relatives (%)	70.5	67.5	82.4	78.8	0.556
Smoking (%)	21.7	27.5	35.3	27.3	0.743
Maternal birth weight (g)*	3429 ± 617	3406 ± 480	3180 ± 808	2965 ± 757	0.042^(5)^
Maternal birth length (cm)*	48.9 ± 2.2	48.7 ± 1.9	47.6 ± 2.3	47.1 ± 3.5	0.05^(6)^
Maternal ponderal index*	2.8 ± 0.2	2.8 ± 0.2	2.9 ± 0.2	3.0 ± 0.6	0.224

(1) *p *< 0.05 IIB *vs *IA, IB;

(2) *p *< 0.01 IA *vs *IIB;

(3) *p *< 0.05 IIB *vs *IB, IIA, IA; IB *vs *IA;

(4) *p *< 0.01 IA *vs *IIB; IB *vs *IIB; IIA *vs *IIB;

(5) *p *< 0.05 IIB *vs *IB, IA;

(6) *p *< 0.05 IIB *vs *IA.

* Maternal weight, length and ponderal index at birth

**Table 2:** Anthropometric (prepregnancy and at screening) and laboratory characteristics at screening according to glucose tolerance status during pregnancy

***Variables***	***Groups***				***p-value***
	***IA***	***IB***	***IIA***	***IIB***	
Height (meter)	1.65 ± 0.07	1.64 ± 0.05	1.61 ± 0.05	1.60 ± 0.05	0.001^(1)^
Legs length (cm)	76.0 ± 4.8	75.3 ± 4.0	73.3 ± 4.1	73.1 ± 4.1	0.014^(2)^
**BMI**					
Prepregnancy	23.1 ± 4.5	25.8 ± 6.1	27.8 ± 7.6	28.5 ± 6.9	0.001^(3)^
At screening	25.6 ± 4.3	29.3 ± 6.1	31.0 ± 6.9	31.4 ± 6.0	<0.001^(4)^
**Waist**					
Prepregnancy	69.6 ± 5.1	81.5 ± 11.3	90.3 ± 18.6	101.8 ± 15.1	<0.001^(5)^
At screening	95.9 ± 10.1	104.5 ± 11.3	107.6 ± 14.4	108.2 ± 9.8	<0.001^(6)^
**Waist-to-hip ratio**					
Prepregnancy	0.76 ± 0.06	0.76 ± 0.05	0.86 ± 0.11	0.87 ± 0.11	0.040
At screening	0.93 ± 0.07	0.95 ± 0.07	0.97 ± 0.04	0.99 ± 0.07	0.002 ^(7)^
**Systolic blood pressure (mmHg)**					
Prepregnancy	108.4 ± 7.8	115.5 ± 23.3	114.7 ± 14.1	125.4 ± 25.8	0.003^(8)^
At screening	107.7 ± 9.9	111.0 ± 12.5	114.7 ± 11.7	120.6 ± 17.6	0.002^(9)^
**Diastolic blood pressure (mmHg)**					
Prepregnancy	71.0 ± 8.2	75.0 ± 14.1	75.8 ± 9.3	81.2 ± 12.9	0.003^(10)^
At screening	71.5 ± 9.2	74.5 ± 10.8	76.4 ± 11.1	79.3 ± 12.2	0.02^(11)^
**At screening**					
Fasting glucose(mg/dl)	73.7 ± 8.7	74.8 ± 9.6	87.2 ± 11.7	114.4 ± 23.6	<0.001^(12)^
HbA_1c _(%)	4.57 ± 0.45	4.95 ± 0.59	5.15 ± 0.71	5.98 ± 1.00	<0.001^(13)^
Total cholesterol(mg/dl)	229.2 ± 43.5	236.7 ± 44.5	221.4 ± 42.3	195.5 ± 43.0	0.001^(14)^
LDL cholesterol(mg/dl)	124.2 ± 41.5	130.2 ± 37.8	111.6 ± 34.6	94.2 ± 33.0	0.001^(15)^
HDL cholesterol(mg/dl)	69.0 ± 16.9	66.1 ± 16.4	73.1 ± 21.3	57.8 ± 16.2	0.014^(16)^
VLDL cholesterol(mg/dl)	34.8 ± 12.0	39.4 ± 13.3	36.5 ± 13.9	43.4 ± 11.7	0.037^(17)^
Triglycerides (mg/dl)	174.3 ± 60.4	215.7 ± 131.7	182.8 ± 69.5	217.3 ± 58.5	0.016^(18)^

(1) *p *< 0.05 IIB *vs *IB, IA; IIA *vs *IA;

(2) *p *< 0.05 IA *vs *IIB, IIA, IB;

(3) *p *< 0.05 IA *vs *IIA, IIB;

(4) *p *< 0.05 IA *vs *IB, IIA, IIB;

(5) *p *< 0.05 IA *vs *IB, IIA, IIB;

(6) *p *< 0.05 IA *vs *IB, IIA, IIB;

(7) *p *< 0.05 IA *vs *IIA, IIB; IB *vs *IIB;

(8) *p *< 0.01 IA *vs *IIB;

(9) *p *< 0.01 IA *vs *IIB;

(10) *p *< 0.01 IA *vs *IIB;

(11) *p *< 0.05 IA *vs *IIB;

(12) *p *< 0.001 IA *vs *IIB, IB *vs *IIB; *p *< 0.01 IA *vs *IIA; *p *< 0.05 IB *vs *IIA, IIA *vs *IIB;

(13) *p *< 0.001 IA *vs *IIB; IB *vs *IIB;

(14) *p *< 0.05 IIB *vs *IIA, IA, IB;

(15) *p *< 0.05 IIB *vs *IA, IB;

(16) *p *< 0.05 IIB *vs *IA, IIA;

(17) *p *< 0.05 IA *vs *IIB;

(18) *p *< 0.05 IA *vs *IIB;

The results of HOMA are shown in Figure 1. The overt diabetes group showed significant increase in HOMA-IR values compared with control group (Fig. 1A; *p *< 0.05). They also exhibited decreased β-cell function, and reduced peripheral insulin sensitivity (as judged by the %β and %S, respectively), when compared to the normoglycemic group (Fig. 1B,C, respectively; *p *< 0.05).

**Figure 1:**
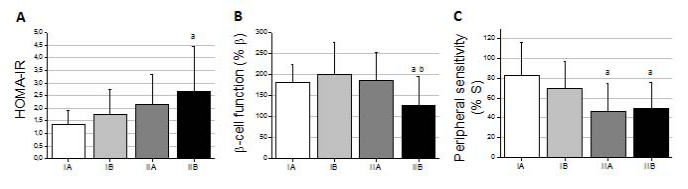
**Evaluation of peripheral insulin sensitivity and β-cell function in patients by HOMA2 model**. These graphs show: **(A) **significant increase of HOMA-IR values **(A)**, decreased β-cell-function **(B)**, and decreased peripheral insulin sensitivity **(C) **in IIB group when compared with IA group. IIA group of patients also exhibited decreased peripheral insulin sensitivity, compared with IA group **(C)**. The values are mean ± SD. ^a^significantly different *vs *IA; ^b^*vs *IB. *p *< 0.05.

There was a significantly increasing prevalence of MS from the control group until the overt GDM group: 0.00%, 20.0%, 23.5%, 36.4% (*p *< 0.001 IA *vs *IB, IIA, IIB). The prepregnancy independent predictors of MS during pregnancy were: previous history of GDM with insulin use OR = 12.90 [95% CI (1.39-119.76)] (*p *= 0.02), BMI > 25 OR = 11.00 [95% CI (4.12-28.95)] (*p *< 0.001), hypertension OR = 10.04 [95% CI (3.25-37.97)] (*p *< 0.001), previous history of GDM without insulin use OR = 6.08 [95% CI (1.66-22.21)] (*p *< 0.001), diabetes in first degree relatives OR = 3.70 [95% CI (1.20-11.38)] (*p *= 0.02), non-Caucasian ethnicity OR = 2.94 [95% CI (1.23-6.67)] (*p *= 0.02), history of prematurity OR = 2.84 [95% CI (1.06-7.57)] (*p *= 0.03) and of polihydramnios OR = 2.57 [95% CI (1.17-5.62)] (*p *= 0.02).

Acanthosis nigricans was the most prevalent physical marker of insulin resistance at screening (19.6, 42.5, 58.8 and 69.7%; for IA, IB, IIA and IIB groups, respectively) (*p *< 0.001 IA *vs *IB, IIA, IIB); followed by obesity (13.0, 40.0, 58.8 and 60.6%; for IA, IB, IIA and IIB groups, respectively) (*p *< 0.001 IA *vs *IIA, IIB); hypertension (6.5, 17.5, 17.6 and 39.4%; for IA, IB, IIA and IIB groups, respectively) (*p *< 0.005 IA vs IB, IIA, IIB), and large waist circumference (2.2, 15.0, 35.4 and 24.2%; for IA, IB, IIA and IIB groups, respectively) (*p *< 0.05 IA *vs *IB; IB *vs *IIB; *p *< 0.01 IA *vs *IIA, IIB).

Independently of glucose tolerance status, the whole group was classified as having or not having MS, and the prevalence of adverse maternal and perinatal outcomes were analyzed. Women that presented MS showed a significant association with the occurrence of preeclampsia OR = 4.08 [95% CI (1.08-15.40)] (*p *< 0.0001). The offspring of mothers with MS presented significantly higher prevalence of LGA OR = 2.97 [95% CI (1.04-8.47)] (*p *= 0.0409), overweight (ponderal index) OR = 4.08 [95% CI (1.65-10.18)] (*p *= 0.001), Apgar score < 7 at 1 min OR = 6.06 [95% CI (1.45-26.94)] (*p *= 0.0083) and 2 min OR = 4.81 [95% CI (1.21-19.09)] (*p *= 0.041), prematurity OR = 3.37 [95% CI (1.30-8.71)] (*p *= 0.031), and any kind of adverse perinatal outcomes OR = 2.88 [95% CI (1.22-6.84)] (*p *= 0.0135).

## Discussion

There is an association between the worsening of glucose tolerance with an increasing prevalence of MS. The worsening of glucose tolerance was directly associated with age [30], non-Caucasian ethnicity, lower education levels, lower family income [31], lower weight and length at birth [32], lower height and shorter legs in adulthood [33], higher BMI, larger waist, larger waist-to-hip ratio [28,34], higher systolic, and diastolic blood pressure [35]. Laboratory findings of lower HDL cholesterol and higher levels of VLDL cholesterol and triglycerides were also associated with the worsening of glucose tolerance. Many of these features are components of MS. This led us to explore the metabolic nature of mild gestational hyperglycemia and GDM as part of the insulin resistance syndrome.

Using the HOMA2 model to assess beta cell function and peripheral insulin sensitivity, we have found that pregnant women that presented any degree of glucose intolerance also showed higher degree of insulin resistance at screening. Patients that presented mild hyperglycemia (impaired glycemic profile and normal OGTT) showed higher values of HOMA-IR and lower peripheral insulin sensitivity, although not statistically significant, compared with the control group, showing that besides presenting insulin resistance, they also had a compensatory hyperinsulinemia. The GDM group (impaired OGTT and normal glycemic profile) had high HOMA-IR values, which were not also statistically significant; however peripheral insulin sensitivity was significantly decreased, pointing to the presence of some insulin action deficiency. Finally, the overt GDM group (both impaired tests) showed the highest values of HOMA-IR probably due to a decreased insulin peripheral action and impaired β-cell function, what points to the presence of both pathophysiological mechanisms that are present in GDM as well as in DM2: insulin resistance and insulin deficiency (Figure 1).

Our study was limited by the small number of participants; so more research is warranted analyzing more patients.

## Conclusion

In conclusion, our data support: that the prevalence of MS increases with the worsening of glucose tolerance; the important role of glycemic profile as a diagnostic test to identify metabolic abnormalities related to MS in pregnancy even in the presence of a normal OGTT, in patients that are not currently classified as having GDM.

## Abbreviations

BMI: Body Mass Index; DM2: type 2 diabetes mellitus; GDM: Gestational Diabetes Mellitus; HOMA: Homeostasis Model Assessment; MS: Metabolic Syndrome; OGTT: Oral Glucose Tolerance Test.

## Competing interests

The authors declare that they have no competing interests.

## Authors' contributions

C.A.N., L.J., A.R. and M.V.C.R participated in the design of the study. C.A.N., M.A.T., B.G., A.D. and M.V.C.R performed the data collection. A.R. and A.D. performed the statistical analysis. C.A.N. and A.R. wrote the paper. All authors read and approved the final manuscript.
